# Clinical Characteristics and Antimicrobial Susceptibility of *Mycobacterium intracellulare* and *Mycobacterium abscessus* Pulmonary Diseases: A Retrospective Study

**DOI:** 10.1155/2022/2642200

**Published:** 2022-01-07

**Authors:** Dongping Wang, Wenhong Lin, Hongyan Cheng, Xundi Bao, Dongfang Xu, Suo Liang, Yue Jiang, Chao Wang

**Affiliations:** ^1^Department of Microbiology, Anhui Chest Hospital, Hefei, China; ^2^Department of Tuberculosis, Anhui Chest Hospital, Hefei, China

## Abstract

The incidence of nontuberculous mycobacteria (NTM) diseases is increasing every year. The present study was performed to investigate the clinical characteristics, CT findings, and drug susceptibility test (DST) results of patients diagnosed with *M. intracellulare* or *M. abscessus* nontuberculous mycobacterial pulmonary disease (NTMPD). This retrospective study included patients diagnosed with NTMPD due to *M. intracellulare* or *M. abscessus* for the first time at Anhui Chest Hospital between 01/2019 and 12/2021. The patients were grouped as *M. intracellulare-*NTMPD group or *M. abscessus-*NTMPD group. Clinical features, imaging data and DST data, were collected. Patients with *M. intracellulare* infection had a higher rate of acid-fast smears (66.1% vs. 45.2%, *P*=0.032) and a higher rate of cavitation based on pulmonary imaging (49.6% vs. 19.4%, *P*=0.002) than patients with *M. abscessus* infection, but both groups had negative TB-RNA and GeneXpert results, with no other characteristics significant differences. The results of DST showed that *M. intracellulare* had high susceptibility rate to moxifloxacin (95.9%), amikacin (90.1%), clarithromycin (91.7%), and rifabutin (90.1%). *M. abscessus* had the highest susceptibility rate to amikacin (71.0%) and clarithromycin (71.0%). The clinical features of *M. intracellulare* pneumopathy and *M. abscessus* pneumopathy are highly similar. It may be easily misdiagnosed, and therefore, early strain identification is necessary. *M. intracellulare* has a high susceptibility rate to moxifloxacin, amikacin, clarithromycin, and rifabutin, while *M. abscessus* has the highest susceptibility rate to amikacin and clarithromycin. This study provides an important clinical basis for improving the management of NTMPD.

## 1. Introduction

The incidence of nontuberculous mycobacteria (NTM) diseases is increasing every year, and it has become a public health concern due to the difficulty of diagnosis, the long course and high cost of treatment, and resistance to most antimicrobial drugs [1–4]. Over 90 species of NTM have been identified in humans [5]. NTM can be found in drinking water systems [6], domestic plumbing, showerheads, and potting soil [7–9]. The estimated NTM prevalence increased from 2.4 per 100,000 persons in 1980 to 15.2 per 100,000 persons in 2013 in the United States of America [10]. Similar figures are observed in Canada [11], the United Kingdom [12], Denmark [13], and Germany [14]. The results of three consecutive epidemiological sampling surveys in China in 1990, 2000, and 2010 showed that the isolation rate of NTM was gradually increasing with 4.9%, 11.1%, and 22.9%, respectively [15]. NTM disease is clinically similar to tuberculosis and therefore can be misdiagnosed as such in the absence of microbiological identification [3]. Besides, most NTM are resistant to antimycobacterial drugs, resulting in poor efficacy of such drugs [16–20].

Among all types of NTM disease, nontuberculous mycobacterial pulmonary disease (NTMPD) is the most common one [21, 22]. In Western countries, the prevalence of NTMPD is higher than the prevalence of tuberculosis [23]. In the United States of America, *Mycobacterium kasassii* is the second most common cause of pulmonary infections, and those bacteria are also responsible for infections in the United Kingdom [2, 24]. *Mycobacterium intracellulare* is most isolated in China, with an isolation rate up to 40%–60% in Northern China [25, 26]. In Southern China, *Mycobacterium abscessus* is also highly isolated in addition to *Mycobacterium intracellulare* [25, 26]. Still, a comprehensive analysis of the clinical characteristics, computed tomography (CT) findings, and drug susceptibility testing (DST) results of *M. intracellulare* and *M. abscessus* has been rarely reported in pulmonary disease.

Therefore, this study aimed to investigate the clinical characteristics, CT findings, and DST results of NTMPD patients diagnosed with *M. intracellulare* or *M. abscessus* infection at their first visit. The results of this study are expected to enrich the epidemiological data of NTM diseases and facilitate the early diagnosis and treatment of NTMPD.

## 2. Materials and Methods

### 2.1. Study Design and Patients

This retrospective study included patients with NTMPD who were first diagnosed with *M. intracellulare* infection or *M. abscessus* infection at Anhui Chest Hospital between January 2019 and December 2021. The study was approved by the ethics committee of Anhui Chest Hospital (approval number: K2021-007). Informed consent was obtained from the patients.

NTM strains were isolated from 772 inpatients during the three years; then, a total of 633 patients with *M. intracellulare* or *M. abscessus* isolated were initially included in the study for further research, and eventually 152 patients were analysed, of whom 121 had *M. intracellulare* pulmonary disease, and 31 had *M. abscessus* pulmonary disease.

All included patients had complete clinical data, appropriate laboratory findings, and imaging findings. Exclusion criteria were (1) NTM infection but no NTM disease, including tuberculosis [16], tumors, COPD, and chronic lung diseases such as bronchial and other diseases; (2) loss of follow-up after no definite diagnosis during hospitalization; (3) diagnosis of *Mycobacterium tuberculosis* lung disease complicated by NTM lung disease; and (4) diagnosis of NTM lung disease and readmission.

The diagnostic criteria of NTMPD are (1) samples not contaminated by exogenous factors; (2) there are respiratory and/or systemic symptoms; (3) there are spongy shadows, multifocal bronchiectasis, and multiple small nodular lesions on chest CT; and (4) two consecutive sputum *Mycobacterium* cultures showing NTM of the same pathogen or one bronchial lavage fluid culture showing a positive result for NTM (2+ or 3+), or a lung tissue biopsy showing histopathological features of mycosis (positive granulomatous inflammation or positive acid-fast staining results) and positive NTM culture results, or biopsies showing histopathological features of the mycobacterial disease (positive granulomatous inflammation or positive acid-fast staining results) and positive NTM culture results in at least one of the sputum and bronchial lavage samples [16, 27].

### 2.2. NTM Culture and Species Identification

**Figure 1:**
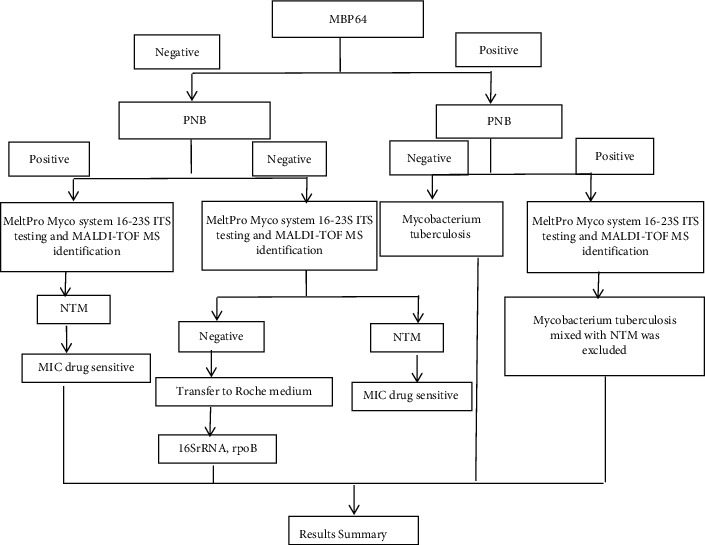
Culture and identification process of NTM.

**Figure 2:**
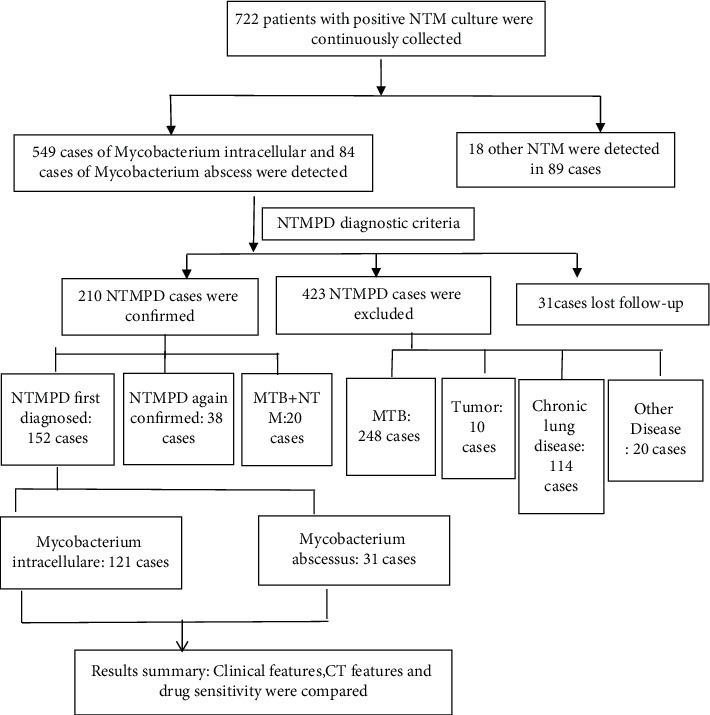
NTMPD sample inclusion.

**Figure 3:**
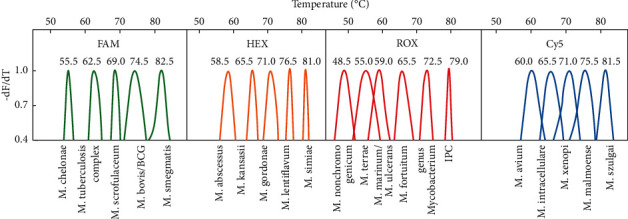
Melting peaks for each species in the MeltPro Myco assay in line with the 2D label strategy. To visually compare the melting temperature values of different *Mycobacterium* species, the melting curves showing the negative derivative of fluorescence intensity with respect to temperature were first normalized between 0 and 1, and then the data between 0.4 and 1 were plotted. *MeltPro Myco assay*: the MeltPro Myco assay targets the intergenic transcribed spacer (ITS) region between the 16S rRNA and 23S rRNA genes of mycobacteria using a panmycobacterial primer set. The MeltPro Myco assay includes 18 species-specific probes used to identify 17 NTM species and *M. tuberculosis* complex (MTBC), a genus-specific probe used to identify the *Mycobacterium* genus, and a set of primers and probes targeting the uninterrupted 229 bp sequence in the *Mycobacterium bovis* genome to distinguish *Mycobacterium bovis* and bacillus *Calmette-Guérin* (BCG) vaccine from M. tuberculosis, and a fragment of the *Arabidopsis thaliana* sucrose-proton symporter 2 (SUC2) gene serves as an internal positive control (IPC) [28]. In total, 21 labels were assigned to 19 mycobacterial species, the genus *Mycobacterium*, and IPC (Figure 3).

Sputum and bronchoalveolar lavage fluid samples were pretreated according to the Practice for Laboratory Testing of Tuberculosis [17] and cultured using the BACTEC MGIT 960 liquid culture method (Supplementary Table 1, and Figures 1 and 2). Strains with positive results by the colloidal gold method in the PNB identification medium (Zhuhai Baso Biotechnology Co., Ltd.) were initially identified using the MPB64 antigen detection kit (Hangzhou Genesis Biodetection & Biocontrol Co., Ltd.). If the bacteria grew in the PNB medium or the MPB64 antigen test showed negative, the strain was tentatively identified as NTM (Figure 3). The strain preliminarily identified as NTM was then identified to species using the MeltPro Myco assay, which targets the intergenic transcribed spacer (ITS) region between the 16S rRNA and 23S rRNA genes of mycobacteria using a panmycobacterial primer set (Zeesan Biotech, Xiamen, China) [28] and the Bruker MALDI-TOF MS identifier [29, 30]. Six technicians with 6 to 15 years of experience, including two deputy chief technicians and four chief technicians, performed the NTM culture and species identification in this study.

### 2.3. DST of NTM

Broth microdilution method was performed according to Clinical and Laboratory Standards Institute (CLSI) guidelines [31, 32], using 96-well round bottom microtiter plates. Microplates and the microplate microbial susceptibility reader for mycobacteria (YK-909, Zhuhai Encode Medical Engineering Co., Ltd.) were used to test the antimicrobial susceptibility of the samples to 14 drugs, including amikacin (1–64 *µ*g/mL), cefoxitin (4–160 *µ*g/mL), clarithromycin (0.5–64 *µ*g/mL), doxycycline (0.5–128 *µ*g/mL), tobramycin (0.5–64 *µ*g/mL), azithromycin (1–32 *µ*g/mL), rifabutin (0.5–32 *µ*g/mL), minocycline (0.5–128 *µ*g/mL), linezolid (0.5–32 *µ*g/mL), rifampicin (1–16 *µ*g/mL), gatifloxacin (0.06–8 *µ*g/mL), ethambutol (2.5–20 *µ*g/mL), moxifloxacin (0.125–16 *µ*g/mL), and compound sulfamethoxazole (8–256 *µ*g/mL). The results were read according to the instructions on the microplate NTM DST kit (Zhuhai Encode Medical Engineering Co., Ltd.). The breakpoint of the minimum inhibitory concentration (MIC) of different drugs were read according to the rules listed in reference [31, 33–35], and CLSI M24-A2 [32] breakpoints were used for interpretation. The quality control strains were ATCC 927, ATCC 27853, and ATCC 29213. Three chief technicians with 10–12 years of experience performed the DST of NTM in the present study according to the previous study [32].

### 2.4. Data Collection

All data were collected from the electronic medical record system (Winning Health CIS 5.5.0.10). Patient demographic and clinical data were collected, including age, gender, underlying disease, comorbidities, family history, medication history, history of antituberculosis treatment, clinical symptoms, laboratory findings, imaging findings (lesion sites and morphological features), and DST results.

### 2.5. Statistical Analysis

SPSS 25.0 (IBM, Armonk, NY, USA) was used for statistical analysis. Continuous data that conformed to a normal distribution (according to the Shapiro–Wilk test) are expressed using means ± standard deviations and were analysed using Student's *t*-test. Data that did not conform to a normal distribution were expressed as “medians (ranges)” and were analysed using the Mann–Whitney *U*-test. Categorical data were expressed as “*n* (%)” and were analysed using the chi-square test or Fisher's exact test. A two-sided *P* value <0.05 was considered statistically significant.

## 3. Results

### 3.1. Clinical Characteristics of the Patients

A total of 633 patients with *M. intracellulare* or *M. abscessus* isolated were first identified, but 481 were excluded: 38 of them were diagnosed with NTMPD at the return visit, 20 with mixed *Mycobacterium tuberculosis* and NTM infection, 423 with NTM infection but no NTM disease, 248 of them were diagnosed with *Mycobacterium tuberculosis* infection, ten had neoplasms, 114 were diagnosed with chronic obstructive pulmonary disease, bronchiectasis, or other chronic pulmonary diseases, and 20 diagnosed with diseases other than NTMPD. In addition, 31 patients who were not diagnosed and lost to follow-up during hospitalization were excluded. Eventually, 152 patients were included, 121 of them had *M. intracellulare* pulmonary disease, and 31 had *M. abscessus* pulmonary disease.

Patients with *M. intracellulare* pulmonary disease had a higher rate of positive acid-fast smears than those with *M. abscessus* pulmonary disease (66.1% vs. 45.2%, *P*=0.032). However, their TB-RNA and GeneXpert results were negative. A similar proportion (62.8% vs. 64.5%) of patients with *M. intracellulare* and *M. abscessus* pulmonary disease received antituberculosis treatment for 1 to 36 months prior to diagnosis, with a mean median antituberculosis duration of 4 months and 6 months, respectively. There were no significant differences between the two groups in terms of age, sex, comorbidities, clinical symptoms, previous antituberculosis treatment, mean duration of antituberculosis treatment, and laboratory findings (all *P* > 0.05) (Table 1).

### 3.2. Pulmonary Imaging Features

The comparison of the imaging findings between the two groups of patients is shown in Table 2. Patients with *M. intracellulare* lung disease had a higher rate of cavitation than patients with *M. abscessus* lung disease according to pulmonary imaging findings (49.6% vs. 19.4%, *P*=0.002). There were no significant differences between the two groups in terms of lesion location, imaging findings of the lesions, mediastinal lymph node enlargement or calcification, emphysema, alveoli, lung injury, and bronchodilation (all *P* > 0.05).

### 3.3. DST Results and Susceptibility Rates

The strains of *M. intracellulare* showed high susceptibility rate to moxifloxacin (95.9%), amikacin (90.1%), clarithromycin (91.7%), and rifabutin (90.1%). The strains of *M. abscessus* showed the highest susceptibility to amikacin (71.0%) and clarithromycin (71.0%).  Table 3 shows the MIC range, MIC 50, and MIC 90 in the two groups.

## 4. Discussion

The incidence of NTM disease is increasing year by year, but analysis of the clinical features, CT findings, and DST results of Chinese patients has rarely been reported. The results of this study indicate that the clinical features of *M. intracellulare* pneumopathy and *M. abscessus* pneumopathy are highly similar, necessitating the identification of molecular biology strains. DST results showed that *M. intracellulare* was highly sensitive to moxifloxacin, amikacin, clarithromycin, and rifabutin, while *M. abscessus* had the highest sensitivity to amikacin and clarithromycin.

Studies indicated that it takes a long time to diagnose NTMPD (7 to 8 years on average), and most patients were first misdiagnosed with pulmonary tuberculosis (92.8%) [36, 37]. A study in Iran showed that among 714 positive acid-fast bacilli from TB-suspected cases, 95 isolates were identified as NTM (13.3%) [38]. Furthermore, many NTMPD patients were infected by a wide range of atypical mycobacteria [39]. Therefore, it is of great importance to differentiate NTM from *Mycobacterium tuberculosis* and to make reliable identification in clinical practice to reduce misdiagnosis and improve treatment efficiency. Before diagnosis, most patients had received repeated antituberculous drug treatment. Our research indicated that most patients were misdiagnosed before they were diagnosed with NTMPD as nearly 70% of the patients in both groups had a history of antituberculosis treatment, which lasted for 4 to 5 months on an average and 3 years on the longest, which was consistent with the previous study. The high rate of misdiagnosis of NTMPD, the difficulty in confirming the diagnosis, and the high cost of treatment when patients are misdiagnosed make it important to find diagnostic markers for NTMPD.

In the present study, out of 633 patients infected with *M. intracellulare* or *M. abscessus*, only 152 were diagnosed with NTM disease at the first visit, 38 were diagnosed with NTMPD at the second visit, 20 were diagnosed with mixed infection with *Mycobacterium tuberculosis* and NTM, 31 were not diagnosed and lost to contact, and the remaining 423 were excluded due to the absence of NTMPD. Such results suggest that NTM strains isolated in the laboratory only prove the possibility of NTM infection. Clinicians should still identify NTMPD based on diagnostic criteria.

Patients of both groups had a high positive rate of acid-fast smears. Those with *M. intracellulare* pulmonary disease having a positive rate of 66.1%, and those with *M. abscessus* pulmonary disease showing 45.2%, which is in agreement with the findings of Riello et al. [40]. However, TB-RNA and GeneXpert results were negative in both groups, suggesting that if a patient has a positive acid-fast smear and a negative TB-RNA and GeneXpert result, then he/she may have NTM disease.

At imaging, lesions of NTMPD are various and diffuse, and double lung lesions are more common in NTMPD than in pulmonary tuberculosis [16, 26, 41, 42]. In this study, patients with *M. abscessus* pulmonary disease had a higher rate of cavitation than patients with *M. intracellulare* pulmonary disease based on lung imaging findings (49.6% vs. 19.4%, *P*=0.002). There were no significant differences between the two groups in lesion location, lesion severity, the characteristics of pulmonary shadow, pleural thickening or pleural effusion, and bronchiectasis, which was in line with the results of a previous study [26].

Most NTM are resistant to traditional antituberculosis drugs, and their resistance patterns vary greatly with different strains. The therapeutic effect after infection mainly depends on the strains identified [43–45].^.^ The consensus of experts on diagnosis and treatment of nontuberculous mycobacterium disease [16] clearly points out that drug sensitivity test before treatment is still very important, and the consensus believes that the correlation between drug sensitivity test results and clinical effect is still difficult to determine, but it is still suggested to base the drug sensitivity results and medication history as far as possible when making chemotherapy regimen for NTM disease. *M. intracellulare* belongs to slowly growing mycobacteria (SGM) of nontuberculous mycobacteria. Expert consensus [16] and An Official ATS/ERS/ESCMID/IDSA Clinical Practice Guideline [27] suggested that macrolides are -the only effective antibacterial agents for the treatment of *Mycobacterium avium*-*intracellulare* infection, and the recommended drugs include clarithromycin, azithromycin, amikacin, rifabutin, rifampicin, and ethambutol. The results of drug sensitivity test in this study showed that the drug sensitivity rate of Mycobacterium intracellulare was clarithromycin (91.7%), amikacin (90.1%), rifabutin (90.1%), rifampicin (82.6%), ethambutol (50.4%), azithromycin (18.2%), and moxifloxacin (95.9%), respectively. The authors of [31, 46–48] reported the drug sensitive rate of clarithromycin (94.2–97.3%), amikacin (57.7–99.4%), rifabutin (95.3%), rifampicin (34.3–75.4%), ethambutol (8.7–75.1%), azithromycin (85.5%), and moxifloxacin (5.5–89.6%). Except azithromycin, the drug sensitivity results in this study were in good agreement with literature reports. In this study, the sensitivity rate of azithromycin (18.2%) was low, which was significantly different from that reported in the literature. The results for clarithromycin predict those for azithromycin, for which testing is problematic as a result of poor solubility at the high concentrations of drug that must be used [32]. The susceptibility rate of clinical *Mycobacterium intracellulare* isolates to moxifloxacin was 95.9%. Wang et al. [46] reported that fluoroquinolone moxifloxacin also had good antibacterial activity against *Mycobacterium intracellulare* in vitro. This study was consistent with literature reports, but moxifloxacin was not among the consensus-recommended drugs.

*M. abscessus* belongs to rapidly growing mycobacteria (RGM) of nontuberculous mycobacteria. *M. abscessus* occupies the largest proportion of RGM that is naturally resistant to and possess acquired resistance to most commonly used antibiotics, commonly treated with antituberculous drugs; also, *M. abscessus* is known as a nightmare bacterium. Expert consensus [16] and An Official ATS/ERS/ESCMID/IDSA Clinical Practice Guideline [27] suggested that the treatment of *M. cheloniae* abscess includes clarithromycin, azithromycin, amikacin, and cefoxitin. The drug sensitivity test results of this study showed that the drug sensitivity rate of *M. abscessus* was clarithromycin (71.0%),azithromycin (32.3%), amikacin (71.0%), linezolid (51.6%), and cefoxitin (67.7%). There are great differences in laboratory drug susceptibility data around the world. The authors of [31, 49–52] reported the susceptibility rates of *M. abscessus to* clarithromycin (62–75.5%),azithromycin (95%), amicacin (15.4–100%), cefoxitin (0–54%), linezolid (14.7–98.1%). In this study, the in vitro antibacterial activities of amikacin (71.0%) and clarithromycin (71.0%) were similar to those reported in [31]. Some natural drug resistance mechanisms of *Mycobacterium abscessus* include waxy and opaque cell walls, drug delivery system, antibiotic modification/inactivation enzyme, and genetic polymorphism of target genes [53]. Macrolides are basic drugs for the treatment of *Mycobacterium intracellulare* and *Mycobacterium abscessus*, and clinical isolates can detect erM(41) (induced macrolide resistance) and/or 23S rRNA point mutation (constitutive macrolide resistance) information, which can obtain the sensitivity of macrolides [51, 53].

This study has limitations. First, it studied only two NTM and had a small sample size so that the results might be biased. Second, due to limited testing competence and methodology, the study did not investigate the resistance-related genetics.

## 5. Conclusions

In conclusion, there are still many difficulties in diagnosing and treating NTMPD, and NTM disease can be easily misdiagnosed. Elderly people are more likely to have NTMPD, and delayed diagnosis of NTMPD and improper antituberculosis treatment are common in clinical practice due to highly similar clinical symptoms and signs, laboratory examinations, and imaging. *M. intracellulare* has a high susceptibility rate to moxifloxacin, amikacin, clarithromycin, and rifabutin, while *M. abscessus* has the highest susceptibility rate to amikacin and clarithromycin.

## Figures and Tables

**Table 1:** Characteristics of the patients with NTMPD.

	*Mycobacterium intracellulare*	*Mycobacterium abscessus*	*P* value
	*n* = 121	*n* = 31	
Age, years, mean ± SD	65.0 ± 10.2	64.7 ± 11.6	0.893
Sex (%)			0.936
Male	65.3 (79/121)	64.5 (20/31)	
Female	34.7 (42/121)	35.5 (11/31)	

*Complications (%)*			
Chronic obstructive pulmonary disease	17.4 (21/121)	9.7 (3/31)	0.296
Bronchiectasis	37.2 (45/121)	51.6 (16/31)	0.397
Diabetes	9.1 (11/121)	16.1 (5/31)	0.322

*Medication history*			
Received antituberculosis treatment before (%)	62.8 (76/121)	64.5 (20/31)	0.861
Mean median antituberculosis duration (months)	4	5	0.974
Glucocorticoid (%)	16.5 (20/121)	19.4 (6/31)	0.709

*Symptoms (%)*			
Cough	71.1 (86/121)	77.4 (24/31)	0.481
Expectoration	61.2 (74/121)	61.3 (19/31)	0.989
Fever	10.7 (13/121)	12.9 (4/31)	0.752
Hemoptysis	15.7 (19/121)	19.4 (6/31)	0.625

*Positive laboratory examination (%)*			
T-SPOT	23.4 (11/47)	20 (2/10)	0.713
Acid-fast smear	66.1 (80/121)	45.2 (14/31)	0.032^*∗*^
Tuberculosis DNA	2.0 (2/102)	6.7 (1/15)	0.34
TB-RNA	0 (0/112)	0 (0/24)	—
Xpert MTB/RIF	0 (0/83)	0 (0/15)	—
Tuberculosis antibodies	55.3 (52/94)	50 (11/22)	0.652
Hemoglobin, g/L, mean ± SD	112.35 ± 27.30	111.00 ± 33.25	0.816
Albumin, g/L, mean ± SD	34.81 ± 4.51	34.47 ± 5.39	0.716
Red blood cells, ×10^12^/L, mean ± SD	4.02 ± 0.66	4.05 ± 0.72	0.824
White blood cells, ×10^9^/L, mean ± SD	5.73 ± 2.09	5.96 ± 2.40	0.591
ESR, mm/h, mean ± SD	32.87 ± 26.11	33.64 ± 29.32	0.852

SD, standard deviation; ESR: erythrocyte sedimentation rate. *P* < 0.05 were deemed statistically significant.

**Table 2:** Pulmonary imaging findings of patients with NTMPD.

Features, *n* (%)	*Mycobacterium intracellulare n* = 58	*Mycobacterium abscessus n* = 16	*P* value
*Site of lesion*			
Left lung	3 (2.5)	0	>0.999
Right lung	7 (5.8)	0	0.346
Both lungs	111 (91.7)	31 (100)	0.215
Cord-like shadow	39 (32.2)	9 (29.0)	0.732
Plaque-like shadow	42 (34.7)	14 (45.2)	0.282
Nodular shadow	45 (37.2)	10 (32.3)	0.61
Pleural thickening or pleural effusion	28 (23.1)	9 (29.0)	0.495
Mediastinal lymph node enlargement or calcification	21 (17.4)	3 (9.7)	0.411
Emphysema, bullae	23 (19.0)	6 (19.4)	0.965
Lung damage	7 (5.8)	1 (3.2)	>0.999
Bronchiectasis	45 (37.2)	16 (51.6)	0.144
Cavity	60 (49.6)	6 (19.4)	0.002^*∗*^

The data are shown as *n* (%). *P* < 0.05 were deemed statistically significant.

**Table 3:** Comparison of DST results and susceptibility rates to 14 drugs in NTMPD.

Drug name, *n* (%)	MIC (*µ*g/mL)			Number (%)		
	MIC range	MIC 50	MIC 90	*S*	*I*	*R*
*Mycobacterium intracellulare, n* *=* *121*						
Amikacin	<1–64	1	16	109 (90.1)	5 (4.1)	7 (5.8)
Azithromycin	1–32	4	32	9 (7.4)	90 (74.4)	22 (18.2)
Clarithromycin	<0.5–64	0.5	64	111 (91.7)	2 (1.7)	8 (6.6)
Linezolid	<0.5–32	8	32	34 (28.1)	68 (56.2)	19 (15.7)
Moxifloxacin	0.125–16	0.5	2	116 (95.9)	3 (2.5)	2 (1.7)
Rifabutin	<0.5–32	0.5	16	109 (90.1)	0	12 (9.9)
Rifampicin	<1–16	1	16	100 (82.6)	8 (6.6)	13 (10.7)
Gatifloxacin	0.06–8	1	4	—	—	—
Doxycycline	4–>128	32	>128	—	—	—
Minocycline	<4–>128	32	64	—	—	—
Sulfamethoxazole	16–>256	192	>256	—	—	—
Cefoxitin	4–160	32	>160	—	—	—
Tobramycin	<0.5–64	4	32	—	—	—
Ethambutol	<2.5–>20	2.5	20	61 (50.4)	33 (27.3)	27 (22.3)

*Mycobacterium abscessus, n* *=* *31*						
Amikacin	<1–64	4	64	22 (71.0)	4 (12.9)	5 (16.1)
Azithromycin	<1–32	16	32	10 (32.3)	6 (19.4)	15 (48.4)
Clarithromycin	<0.5–64	0.5	64	22 (71.0)	2 (6.5)	7 (22.6)
Linezolid	2–32	8	32	16 (51.6)	9 (29.0)	6 (19.4)
Moxifloxacin	0.125–16	4	16	9 (29.0)	7 (22.6)	15 (48.4)
Rifabutin	<0.5–2	2	32	—	—	—
Rifampicin	<1–16	16	16	—	—	—
Gatifloxacin	0.06–8	2	8	—	—	—
Doxycycline	16–>128	>128	>128	0	0	31 (100)
Minocycline	<4–>128	64	>128	—	—	—
Sulfamethoxazole	32–>256	>256	>256	1 (3.2)	1 (3.2)	29 (93.5)
Cefoxitin	4–160	48	96	21 (67.7)	0	10 (32.3)
Tobramycin	<0.5–64	32	64	3 (9.7)	5 (16.1)	23 (74.2)
Ethambutol	<2.5–>20	>20	>20	—	—	—

S, susceptible; I, intermediary; R, resistant. Because there is no recognized breakpoint, the drug resistance rate cannot be calculated.

## Data Availability

All the data generated or analysed during this study are included in this published article.

## Supplementary Materials

Supplementary Materials Supplementary Table 1: sample composition of sputum and bronchoalveolar lavage fluid in the two groups.
