# Supplementary material for: Clinical Characteristics and Antimicrobial Susceptibility of Mycobacterium intracellulare and Mycobacterium abscessus Pulmonary Diseases: A Retrospective Study
Source: Can J Infect Dis Med Microbiol. 2022 Jan 7;2022:2642200. doi: 10.1155/2022/2642200 (PMC8759892; doi:10.1155/2022/2642200)
Supplement: Supplementary Materials — Supplementary Table 1: sample composition of sputum and bronchoalveolar lavage fluid in the two groups. [file 2642200.f1.docx]

Supplementary Table 1. Sample composition of sputum and bronchoalveolar lavage fluid in the two groups

|  | *Mycobacterium intracellulare* n=121 | *Mycobacterium abscessus* n=31 |
| --- | --- | --- |
| phlegm | 93 | 24 |
| BALF | 28 | 7 |
